# Retraction: Sanal Flow Choking: A Paradigm Shift in Computational Fluid Dynamics Code Verification and Diagnosing Detonation and Hemorrhage in Real‐World Fluid‐Flow Systems

**DOI:** 10.1002/gch2.202470082

**Published:** 2024-03-25

V. R. S. Kumar, V. Sankar, N. Chandrasekaran, A. Sukumaran, S. A. R. M. Rafic, R. S. Bharath, R. V. Baskaran, C. Oommen, P. K. Radhakrishnan, S. K. Choudhary, “Sanal Flow Choking: A Paradigm Shift in Computational Fluid Dynamics Code Verification and Diagnosing Detonation and Hemorrhage in Real‐World Fluid‐Flow Systems.” *Global Challenges* 2020, *4*, 2000012. https://doi.org/10.1002/gch2.202000012

The above article, published on May 27, 2020 in Wiley Online Library, has been retracted by agreement between the journal Editor‐in‐Chief, Mara Staffilani, and Wiley‐VCH GmbH, Weinheim.

The retraction has been agreed due to concerns raised by a third party in a submitted Comment regarding the paper's proposed theory and its application of concepts.

Post‐publication review by an independent reviewer determined that the article applies the concept of compressible flow to an inherently incompressible flow system. As a result, the article's hypothesis and following argument do not scientifically support its conclusions. Therefore, the conclusions are considered unreliable.

An investigation by Wiley and the journal's Editor‐in‐Chief supported this conclusion.

